# Supplementary material for: Reactions to macro-level shocks and re-examination of adaptation theory using Big Data
Source: PLoS One. 2024 Jan 31;19(1):e0295896. doi: 10.1371/journal.pone.0295896 (PMC10830054; doi:10.1371/journal.pone.0295896)
Supplement: S2 Appendix — (DOCX) [file pone.0295896.s002.docx]

**S2 Appendix. External validity of the Gross National Happiness index.**

Please note that this validation exercise was part of ongoing research and development and includes information previously published in Sarracino et al. [1].

Assessing the convergent validity of metrics based on unstructured data, such as Twitter data, is difficult because their features - timeliness, large (non-representative) samples, and high frequency - make them unique, thus limiting the availability of comparable measures. In saying this, Garcia et al. [2] compared weekly YouGov survey data to 1.54 billion tweets originating in the UK (June 2019 – June 2021) and found positive and consistent correlations between the survey and Twitter data. Since comparable measures of well-being (objective or subjective) with the same frequency and timeliness as the GNH are scarce, Sarracino et al. [1] correlated the GNH with measures of well-being obtained using other sources of Big Data, such as Google. However, the downside of this approach is that it relies on the assumption that data issued from Google are valid.

Sarracino et al. [1] assess the validity of GNH using the following approaches: firstly, they check whether GNH correlates with survey measures of subjective well-being using cross-sectional country-level data; secondly, they test whether changes in GNH over time correlate significantly with some of the few sources of repeated observations on subjective well-being available in 2020, and with Google data.

**Correlation across countries**

Sarracino et al. [1] first assess the validity of GNH by calculating its correlation with life satisfaction, a widely used measure of subjective well-being whose validity and reliability have been largely confirmed (OECD [3]). Observing a high correlation between GNH and life satisfaction indicates that GNH reflects similar factors affecting life satisfaction and suggests it is a valid measure of subjective well-being.

The measure of life satisfaction is available from Eurobarometer surveys conducted by the European Commission. Eurobarometer surveys have been conducted biennially since the 1980s to measure public opinion in the European Union. Each survey wave is comprised of approximately 1000 face-to-face interviews in each country. Due to the COVID-19 pandemic, many countries could not conduct face-to-face interviews. Respondents answered online and were recruited using a probabilistic method by telephone. See European Commission [4] for additional details. It is measured as the response to the question, "On the whole, are you very satisfied, fairly satisfied, not very satisfied or not at all satisfied with the life you lead?". Response options are coded from one to four, with greater values representing greater satisfaction. Sarracino et al. [1] used the Standard Eurobarometer 93.1 fielded from 9 July to 26 August 2020 in the European Union, the United Kingdom, and five EU candidate countries (European Commission [4]).

S8 Fig depicts the correlation of GNH with life satisfaction. Average GNH is computed by country over the period mid-July to the end of August, i.e., the months when Eurobarometer surveys were administered. The scatterplot indicates that there is a positive association between the two measures. The Spearman correlation coefficient is 0.37 (not statistically significant), which appears as an outlier if Italy is excluded. The correlation coefficient is 0.32, but not statistically significant if Italy is included.


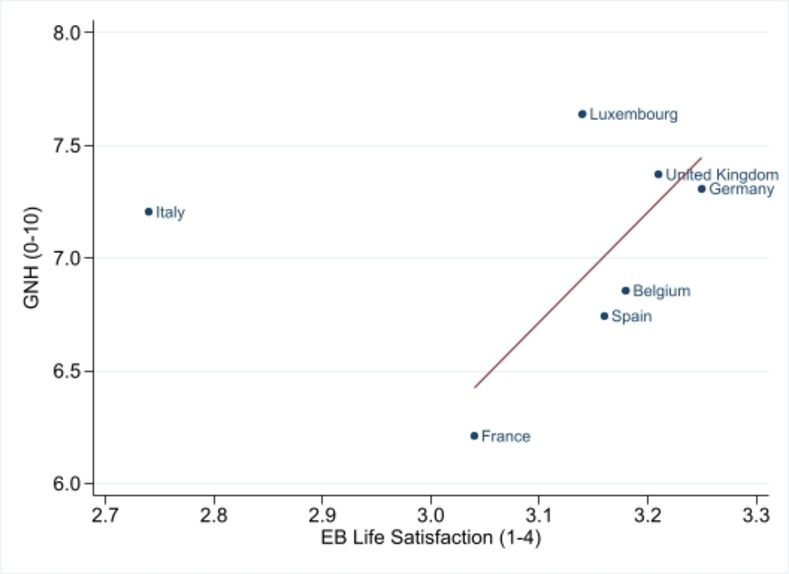


**S8 Fig. Average Gross National Happiness correlates positively with average life satisfaction.**

Note: The GNH score is the average by country over the same period that the Eurobarometer was collected, from 9 July to 26 August 2020.

Source: GNH data (Greyling et al. [5]) and life satisfaction data are from the Eurobarometer (European Commission [4]), Summer 2020.

Average GNH correlates meaningfully with the measure of subjective well-being reported by the World Happiness Report 2021 (Helliwell et al. [6]), i.e., the average life evaluation from 2018 to 2020 (see S9 Fig). The report uses the Gallup World Poll data to rank countries from the happiest to the least happy. The Spearman correlation between GNH and average life evaluation is 48% ($Prob>\left| t \right|=0.16, N=10)$. After excluding South Africa and France, which appear as outliers, the Spearman correlation is 20% ($Prob>\left| t \right|=0.65, N=8$).


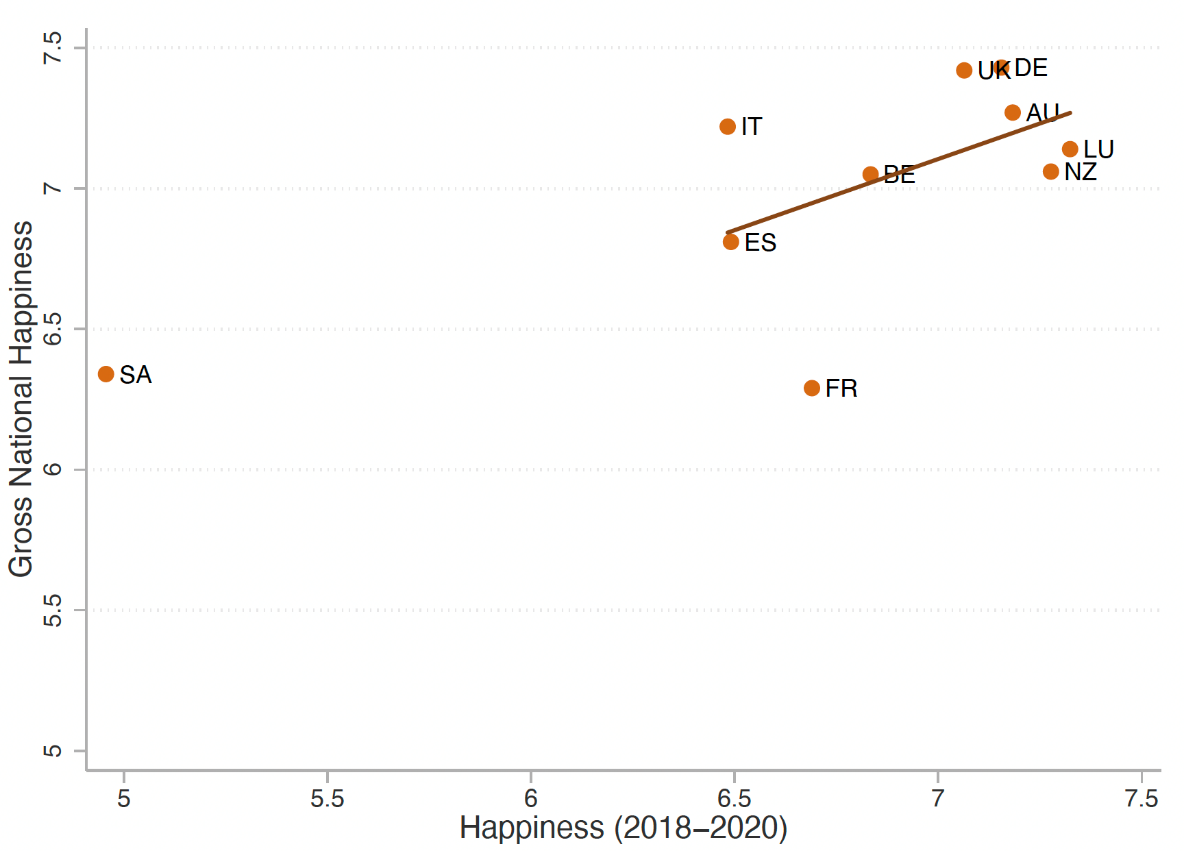


**S9 Fig. Correlation between average GNH in 2020 and average life evaluation (2018-2010) from the World Happiness Report 2021.**

Note: average life evaluation is computed over the years 2018, 2019 and 2020. Average GNH is computed over the year 2020.

Source: GNH data (Greyling et al. [5]) and average life evaluation are sourced from the World Happiness Report, 2021 (Helliwell et al. [6]).

**Correlation over time**

Correlation over time is an important validity test for a measure that has the benefit of timeliness and frequency. For this purpose, Sarracino et al. [1] use two sources of repeated observations on subjective well-being and ill-being for 2020: Google Trends and consumer confidence data provided by Eurostat.

Google Trends is a source of daily search results by country used in numerous research projects ranging from assessing economic conditions to individuals' feelings (see, for example, Brodeur et al. [8]). Rather than focusing on the trends of topics such as "happiness, well-being, or life satisfaction", which may not accurately reflect the well-being of Google users, Sarracino et al. [1] created an index of negative emotions (dashed line in S10 Fig) by averaging daily Google search scores for three topics: fear, sadness, and anger. The correlation coefficient between GNH and the index of negative emotions is -0.27 ($Prob>\left| t \right|=0.39, N=12 months$). The negative sign is to be expected, as the index of negative emotions should correlate negatively with a measure of well-being. A visual inspection of S10 Fig reveals that, indeed, GNH and the index of negative emotions move in the expected direction, as they document worsening well-being over 2020. The main discrepancy is observed for the first half of the year when GNH decreases less and more slowly than the index of negative emotions.


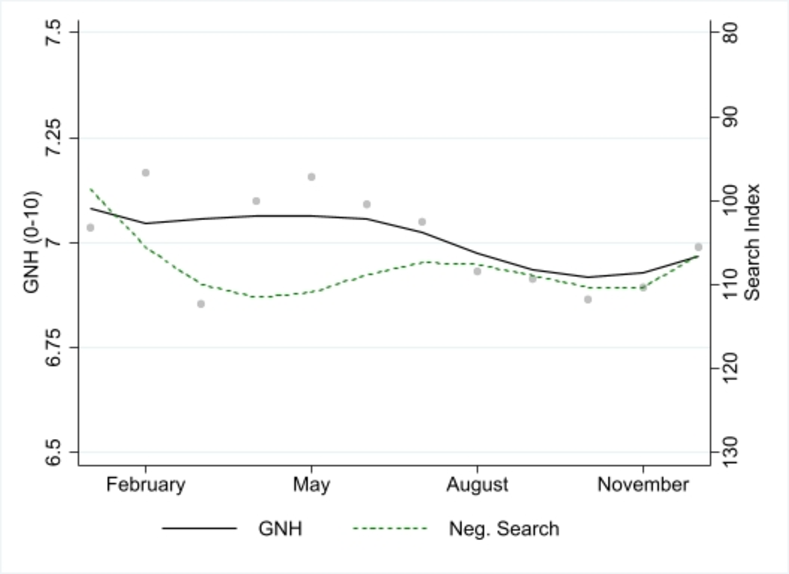


**S10 Fig. Gross National Happiness correlates meaningfully with the index of negative emotions over time.**

Note: The index of negative emotions is the average of weekly averages of negative emotions (fear, sadness and anger) within countries.

Source: GNH data (Greyling et al. [5]). The index of negative emotions is sourced from Google LLC [9].

Finally, Sarracino et al. [1] use consumer confidence data (Eurostat [10]) as a source of repeated observations to validate GNH. Although consumer confidence relates more to the economic and material domain of people's lives, it should positively correlate with GNH and are available at a relatively high frequency. Consumer confidence is monitored via monthly surveys administered by Eurostat to European Union Member States residents. The final score is an index that averages positive and negative feelings of consumers in relation to their economic conditions and perspectives. S11 Fig shows that the monthly changes in GNH correlate positively with the changes in consumer confidence: the Spearman correlation coefficient is 0.5, $(Prob>\left| t \right|=0.17, N=9 months$ ). Please note that three observations are missing because of the initial month, for which Sarracino et al. [1] could not compute the change, and because data for Italy in April are missing. Therefore, it was not possible to compute the changes relative to March and April and April and May.


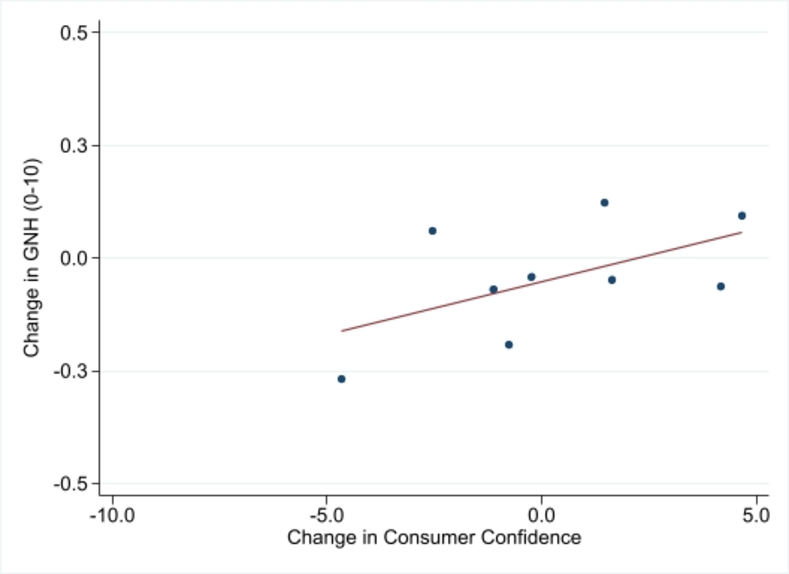


**S11 Fig. Gross National Happiness correlates meaningfully with consumer confidence data.**

Note: Consumer confidence is a monthly index averaging positive and negative feelings about economic conditions and perspectives.

Source: GNH data (Greyling et al. [5]). Consumer confidence data are from the European Commission (Eurostat [10]).

**References**

1. Sarracino F, Greyling T, O'Connor K, Peroni C, & Rossouw S. A year of pandemic: levels, changes and validity of wellbeing data from Twitter. Evidence from ten countries. PLOS ONE. 2023; 18(2): e0275028.
2. Garcia D, Pellert M, Lasser J, & Metzler H. Social media emotion macroscopes reflect emotional experiences in society at large. ArXiv. 2021; 2107:13236.
3. OECD. Guidelines on Measuring Subjective Well-being. 2013. OECD.
4. European Commission. Standard Eurobarometer 93 - public opinion in the European Union, first results, annex. 2020.
5. Greyling T, Rossouw S, & Afstereo. Gross National Happiness Index. 2019. Available from <http://gnh.today>
6. Helliwell JF, Layard R, Sachs J, De Neve JE, editors. World Happiness Report. 2021. Sustainable Development Solutions Network, New York.
7. University of Luxembourg. Come-here (covid-19, mental health, resilience and self-regulation) longitudinal survey. 2020.
8. Brodeur A, Clark AE, Fleche S, & Powdthavee N. COVID-19, lockdowns and well-being: Evidence from Google Trends. Journal of Public Economics. 2021; 193.
9. Google LLC. Google trends. 2021. Accessed: January 2021.
10. Eurostat. Business and consumer surveys (dg ecfin) (ei_bcs) meta- data in euro sdmx metadata structure (esms). 2020a.
