# Supplementary material for: Reactions to macro-level shocks and re-examination of adaptation theory using Big Data
Source: PLoS One. 2024 Jan 31;19(1):e0295896. doi: 10.1371/journal.pone.0295896 (PMC10830054; doi:10.1371/journal.pone.0295896)
Supplement: Attachment — Submitted filename: Comments on PONE-D-22-19460r2.pdf [file pone.0295896.s008.pdf]

Comments on PONE-D-22-19460r2 "Re-examining adaptation theory using Big Data: Reactions to External Shocks"

The authors have responded in detail to my previous points and have put considerable effort into the paper. I remain concerned about a couple of points where, I suspect, we will have to agree to disagree. It will be for the editor to decide how to handle those differences.

The first is the issue of treating lockdown as a 'shock' since it is really COVID that was the shock and the lockdown was a response to that shock. I appreciate that the paper is not the first to do this. Even so, I find it hard to interpret the impact of lockdown with COVID used as independent right-hand side variables.

Second, I think that treating 2021 as a counterfactual period is problematic due to the dynamic and on-going effects of COVID. It's very tricky interpreting results relative to a counterfactual period which is itself unstable.

Third, I'd like to have seen some engagement with the common trends assumption in the paper but there is none despite the fact that it's crucial to dif-in-dif estimation. If there is no common trend then one needs to resort to something to tackle that such as synthetic matched dif-in-dif.

Fourth, I still don't see the value of the North v South analysis. If it's intended to capture intensity of treatment it's a pretty crude way of doing so.

Finally I also remain concerned by the N observations (point 5 in my previous comments) notwithstanding the additional test the authors report.
