# Supplementary material for: Reactions to macro-level shocks and re-examination of adaptation theory using Big Data
Source: PLoS One. 2024 Jan 31;19(1):e0295896. doi: 10.1371/journal.pone.0295896 (PMC10830054; doi:10.1371/journal.pone.0295896)
Supplement: Attachment — Submitted filename: plos one comments.docx [file pone.0295896.s004.docx]

1. The introduction should be rewritten better
   1.1 Long introduction and includes few citations. For example, the part between line 55 and line 85 does not have any citation
   1.2 The part between line 85 and line 98 is more suitable to be in related works
2. From the text, it is not possible to know if the tweets dataset is suitable for this type of study or not.

Authors should add a table showing how tweets are selected for each country. For example, the language and if the tweet has to contain a specific hashtag.

3. Authors have to add more description about the Ml models that they use to predict the sentiment like:

Are the models trained by the authors or not

is there a model for each language

what is the performance of each model

4. Apart from the factors to be studied, the countries that have been selected are not homogeneous, and each country has different events that affect the level of happiness in it. why It is not mentioned in the text anything about that?
