# Supplementary material for: Reactions to macro-level shocks and re-examination of adaptation theory using Big Data
Source: PLoS One. 2024 Jan 31;19(1):e0295896. doi: 10.1371/journal.pone.0295896 (PMC10830054; doi:10.1371/journal.pone.0295896)
Supplement: Attachment — Submitted filename: Response to reviewers (21).docx [file pone.0295896.s005.docx]

RESPONSE TO REVIEWERS

**Manuscript ID PONE-D-22-19460**

**Re-examining adaptation theory using Big Data: Reactions to external shocks.**

We thank the reviewers for the comments. Each has been comprehensively addressed, as shown below (*authors' responses shown in italics*). Throughout the paper, we have also improved its quality and contribution to the literature. Additionally, we have taken onboard all the suggestions made by reviewers #1 and #2 and re-estimated all models with new results in the revised manuscript. Therefore, we believe the paper to be significantly improved due to the comments received from the reviewers.

**Journal requirements addressed by the authors**

When submitting your revision, we need you to address these additional requirements.

**1.**	**Please ensure that your manuscript meets PLOS ONE's style requirements, including those for file naming. The PLOS ONE style templates can be found at**

[**https://journals.plos.org/plosone/s/file?id=wjVg/PLOSOne_formatting_sample_main_body.pdf**](https://journals.plos.org/plosone/s/file?id=wjVg/PLOSOne_formatting_sample_main_body.pdf) **and**

[**https://journals.plos.org/plosone/s/file?id=ba62/PLOSOne_formatting_sample_title_authors_affiliations.pdf**](https://journals.plos.org/plosone/s/file?id=ba62/PLOSOne_formatting_sample_title_authors_affiliations.pdf)

*Thank you for providing the information. Subsequently, all submission file names follow PLOS ONE's requirements.*

**2.	In your Methods section, please include additional information about your dataset and ensure that you have included a statement specifying whether the collection and analysis method complied with the terms and conditions for the source of the data.**

*After incorporating the reviewer's comments, additional information on our dataset has been included. Please note that this is our original data; therefore, no terms and conditions must be complied with.*

**3.	We note that you have referenced (Silver, RLet al. [2]) which has currently not yet been accepted for publication. Please remove this from your References and amend this to state in the body of your manuscript: (ie "Silver, RL et al. [Unpublished]") as detailed online in our guide for authors** [**http://journals.plos.org/plosone/s/submission-guidelines#loc-reference-style**](http://journals.plos.org/plosone/s/submission-guidelines#loc-reference-style)

*Subsequently, we removed the study conducted by Silver from the manuscript.*

**Reviewer comments addressed by the authors.**

**Reviewer #1:**

1. **The introduction should be rewritten better**
   1.1 **Long introduction and includes few citations. For example, the part between line 55 and line 85 does not have any citation**

*We thank the reviewer for this comment. Subsequently, section 1 has been changed. We wish to point out that the section you refer to, which does not have citations, includes our aims, methods and contributions; therefore, there shouldn't be citations.*

1.2 **The part between line 85 and line 98 is more suitable to be in related works**

*We thank the reviewer for this suggestion. Subsequently, this has been shortened, although some of it remains to highlight previous studies and how we contribute to the existing literature.*

2. **From the text, it is not possible to know if the tweets dataset is suitable for this type of study or not.**

**Authors should add a table showing how tweets are selected for each country. For example, the language and if the tweet has to contain a specific hashtag.**

*We thank the reviewer for this comment. Subsequently, section 3.2 was rewritten to explain in detail the process we follow regarding the extraction of tweets. Additionally, we added in this response document under point #4 of reviewer 2 country examples of the GNH accurately reflecting the mood of nations. These add credibility to the GNH being a suitable dataset for happiness and macro-level shocks. The country examples come from the following published work:*

*References*

*Rossouw, S., & Greyling, T. (20200. Big Data and Happiness. In: Zimmermann, K.F. (eds) Handbook of Labor, Human Resources and Population Economics. Springer, Cham.* [*https://doi.org/10.1007/978-3-319-57365-6_183-1*](https://doi.org/10.1007/978-3-319-57365-6_183-1)

3. **Authors have to add more description about the Ml models that they use to predict the sentiment like:**

**Are the models trained by the authors or not is there a model for each language what is the performance of each model?**

*We thank the reviewer for this comment. We used a lexicon-based* (*a lexicon is a library of words that stores the meanings and structures of words) analysis for the sentiment analysis and expanded our methodology in section 3.2. In essence, we use a type of machine learning (Natural Language Processing) as the machine must recognise the words or the sequence of words included in the specific library from the corpus data (tweets), after which it codes the sentiment or emotion.*

*As noted in the manuscript, we used Sentiment140, VADER and the Syuzhet bouquet, which includes five lexicons. Each of these lexicon's methodologies is explained; for example, Sentiment140 uses Naive Bayes classification (*[*https://streamhacker.com/2010/05/10/text-classification-sentiment-analysis-naive-bayes-classifier/*](https://streamhacker.com/2010/05/10/text-classification-sentiment-analysis-naive-bayes-classifier/)*).*

*Other lexicons (such as Syuzhet) use Naïve Bayes, Decision trees, KNN, and SVM classifiers (each lexicon states which classifiers are used). Furthermore, it seems that the rule-based lexicons perform very well using measures such as the Accuracy, Recall, Precision, and F1 Score (see, for example,* [*https://towardsdatascience.com/sentiment-analysis-in-10-minutes-with-rule-based-vader-and-nltk-72067970fb71*](https://towardsdatascience.com/sentiment-analysis-in-10-minutes-with-rule-based-vader-and-nltk-72067970fb71)*).*

*We take advantage of the abovementioned lexicons' ability to be applied to different languages. In the event that we do not have the specific language within the lexicon, we use Microsoft Azure and Google Translate to translate the tweets into English, whereafter they are coded.*

4. **Apart from the factors to be studied, the countries that have been selected are not homogeneous, and each country has different events that affect the level of happiness in it. why It is not mentioned in the text anything about that?**

*We thank the reviewer for this comment. In sections 3.3, 3.4.1 and 3.4.2, we highlight that we include country-fixed effects to control for countries' observed and unobserved characteristics.*

**Reviewer #2:**

**The question the authors are addressing is an interesting and important one, and there are features of there data that are attractive – notably their use of Twitter to capture real-time changes in sentiment – but I was not convinced by the empirical analysis and remain uncertain about the contribution to the literature. It's not entirely clear to me from the descriptions of the data used in the study whether these concerns can be addressed in a revision. I elaborate on these points below.**

**1.	Motivation and Contribution**

**1.1	The motivation and contribution were unclear to me. The authors talk of a contribution at the 'macro' level but, in fact, their analysis is really at the micro level using country fixed effects. There is nothing wrong with that – indeed it's the right way to go empirically – but it means we are focusing on within country variance in sentiment/wellbeing over time, rather than comparing across countries as some might expect given the references to the macro-level in the set up. This probably needs revisiting.**

*We thank the reviewer for this comment. Whereas a single tweet would be seen as representative of the micro-level, we extract all tweets within a bounding box (country). These tweets are coded (via sentiment and emotion analysis) in real-time, and we derive summary statistics per country per day. Our analyses use the time series data per country and not the raw tweets; therefore, our analyses are at the country level (macro). By extension, this implies that our analysis is a between-country rather than a within-country analysis. To remove any possible confusion among readers, we have extensively rewritten section 3.2, in which we elaborate on the construction of the Twitter dataset used in the paper.*

**1.2	As an aside, it was never clear to me why macro effects should differ from an aggregation of the micro-effects in this case. That point also needs discussion if the authors choose to stick to the macro issue as a contribution.**

*We thank the reviewer for this comment. Knowing how happiness changes at the macro level is important for several reasons. From the works done by (Goldenberg et al., 2020), we know that when considering numerous people's interactions, macro-level affective processes occur and cannot be readily captured when one examines the individual level alone. This is because macro-level affective processes differ from the individual-level responses in terms of their quality (reduced variability), magnitude (increased intensity), and time course (prolonged). Such macro-level affective processes seem to contribute to the unfolding of a variety of collective processes driven by both negative emotions (e.g., violent demonstrations, civil unrest, fear, and collective mourning) and positive emotions (e.g., excitement, hope, and collective celebrations such as sport teams winning).*

*Additionally, as Anik et al. (2009) and Lyubomirsky et al. (2005) pointed out, happiness also has macro-level consequences regarding a country's social and health sectors. Happy people display more altruistic behaviour in the long run. They are also more active, creative, better problem solvers and more social while displaying less anti-social behaviour – thereby increasing national social capital. In terms of health, happy individuals are physically healthier, live longer and engage less in risky behaviour, such as smoking and drinking – thereby alleviating pressure on national health resources. Piekalkiewicz (2017) states that happiness may act as a determinant of broader economic outcomes such as an increase in productivity, predicting one's future income and affecting labour market performance.*

*In survey data such as Gallup, a similar method is applied. Surveys are at the micro-level, and responses are aggregated to derive macro-level happiness scores – thus, the concept is not new (Helliwell et al., 2023). Even considering gross domestic product (GDP) is the income per person aggregated to derive national GDP.*

*Lastly, given that the main focus of the World Happiness Reports (Helliwell et al., 2023) lies with macro-level consequences of a decrease in life satisfaction and the Gallup Global Emotions Report (Gallup, 2022) cites the effect of collective emotions (macro-level), such as the relationship between negative emotions and civil unrest, it is increasingly important to interpret macro-level events.*

*References*

*Anik, L., Aknin, L. B., Norton, M. I., & Dunn, E. W. (2009). Feeling Good About Giving: The Benefits (and Costs) of Self-Interested Charitable Behavior. Harvard Business School Marketing Unit Working Paper No. 10-012. Available at SSRN:* [*https://ssrn.com/abstract=1444831*](https://ssrn.com/abstract=1444831)

*Gallup. (2022). Global Emotions Report 2022. Available from* [*https://www.gallup.com/analytics/349280/gallup-global-emotions-report.aspx*](https://www.gallup.com/analytics/349280/gallup-global-emotions-report.aspx)

*Goldenberg, A., Garcia, D., Halperin, E., & Gross, J. J. (2020). Collective emotions. Current Directions in Psychological Science, 29(2), 154–160.*

*Helliwell, J. F., Layard, R., Sachs, J. D., Aknin, L. B., De Neve, J.-E., & Wang, S. (Eds.). (2023). World Happiness Report 2023 (11th ed.). Sustainable Development Solutions Network.*

*Lyubomirsky, S., Sheldon, K. M., & Schkade, D. (2005). Pursuing happiness: The architecture of sustainable change. Review of General Psychology, 9: 111–131.*

*Piekalkiewicz, M. (2017). Why do economists study happiness? The Economic and Labour Relations Review, 28(3):361–77.*

**1.3	I also thought that the review of existing literature on habituation and adaptation was very partial, missing some important contributions from the literature in conflict, war and terrorism, such as the work of Krueger on 9/11 and the work of Clark et al on the Boston Bomber. These and many other papers have established the rapid return to pre-event wellbeing following horrific events such as bombings. I encourage the authors to go back to that literature and re-read it so as to think more carefully as to what their contribution might be. At present I'm not convinced that contribution is sufficient to merit publication.**

*We thank the reviewer for these comments. We have rewritten our motivation and contributions in section 1 accordingly after adding the literature about macro-level shocks and their effect on well-being and emotions in section 2.*

*We now make it clear that our motivation is to compare two shocks with different intents, a government regulation shock with an intent to save lives and a war shock intended to disregard human life. Additionally, we motivate our study by comparing a war shock to a lockdown shock in order to see whether the type of shock plays a role in the effect on happiness. Furthermore, we want to compare the shocks' effect size and determine whether the adaptation period differs across different types of shocks. This has never been done before and significantly contributes to the field of adaptation and macro-level shocks.*

**2.	The characterisation of shocks and the timing of events**

**2.1	The timing of the Ukraine shock as the moment of invasion seems reasonable, notwithstanding some likely anticipation, but the timing of the COVID shock is really the moment the pandemic led to a big increase in infection rates. Yes the infection rate – which arguably captures the intensity of the COVID outbreak and could thus be used to identify before/after periods – is used as a right-hand side control variable in the analyses. This seems odd to me. Instead, the authors focus on the date of lockdowns which are a policy response to the shock, not the shock itself.**

*We thank the reviewer for highlighting this issue. Subsequently, we rewrote the paper to highlight the two macro-level shocks we are investigating. The first shock is the lockdown due to government regulation, with the ultimate negative effect recorded across emotions and economic and other social factors being unanticipated. The second shock was the invasion of Ukraine, which Ukraine anticipated, but the event's timing was unknown. However, the subsequent negative effects which impacted countries not directly involved in the conflict were unanticipated.*

**2.2	If the authors stick with lockdowns as the treatment they need to think hard about whether and why they condition on COVID cases since this nets out much of the health shock. One might want to do that if one is thinking about the effects of lockdowns per se, but that's not really discussed in the paper.**

*We thank the reviewer for this comment. Following on from point #2.1 above, we have clarified that we are analysing the lockdown shock against the background of the COVID-19 pandemic.*

**3.	Dif in Dif Estimation**

**3.1	The real time moment for the Ukraine shock is the same for all countries. But the real time before/after lockdown varies by country by a few weeks. This variance can be important, especially for more granular analysis as in the event study, because the new dif-in-dif literature (which is not referred to in the paper) spends a lot of time discussing how to deal with potential confounding arising from combining different pre- and post-periods in pooled data when the timing of the 'treatment' differs across units. This needs thought and probably further empirical investigation. More broadly there is no discussion of the identification assumptions relating to dif-in-dif and no examination of them, eg. In relation to common trends. There is evidence of some anticipation effects which, again, needs further discussion/examination because that's problematic for dif-in-dif estimation.**

*We thank the reviewer for this comment. Subsequently, we investigated the new DiD methodologies put forth in the studies by de Chaisemartin and d'Haultfoeuille (2020) and Roth et al. (2022).*

*In our study, the timing of the shock is the day of the lockdown announcement, i.e., the start of the treatment and denoted as day 0 for all our countries. Before the announcement, countries had no information on whether or not they would go into lockdown. Therefore, it was a seemingly unanticipated event, with some countries choosing not to lock down their citizens. This is in contrast to an experiment in which the treatment is scheduled to start at a specific time in different test centres, i.e., being planned and expected.*

*In our analysis, a day counter was used to capture time.* *We believe that the differences in the lockdown dates contribute to the identification, as the lockdown event is most likely the cause of the decrease in the GNH rather than other factors (for a similar conclusion, see Brodeur et al. (2021)).*

*We extended the identification assumptions and emphasised them more in section 3.4.1.*

*References*

*Brodeur, A., Clark, A. E., Fleche, S., & Powdthavee, N. (2021). COVID-19, lockdowns and well-being: Evidence from Google Trends. Journal of Public Economics, 193:* *104346.*

*De Chaisemartin, C., d’Haultfoeuille, X. (2020). Two-Way Fixed Effects Estimators with Heterogeneous Treatment Effects. American Economic Review, 110(9), 2964-2996.*

*Roth, J., Sant' Anna, P. H. C., Bilinski, A., & Poe, J. (2022).* [*What's Trending in Difference-in-Differences? A Synthesis of the Recent Econometrics Literature*](https://ideas.repec.org/p/arx/papers/2201.01194.html)*.* [*Papers*](https://ideas.repec.org/s/arx/papers.html) *2201.01194, arXiv.org, revised Jan 2023.*

**3.2	It's hard to think of 2021 as a 'counterfactual' period given the dynamic and on-going effects of COVID. The authors talk of standardising their Tweet sentiment data in a way that they say helps deal with this but I was unable to work out precisely how this standardisation was undertaken and why it would address the issue.**

*We thank the reviewer for this comment. Subsequently, we decided not to standardise (mean=0 and standard deviation = 1) the GNH data. Please note that this decision does not affect the regression results. In an effort to remove any confusion this might create among readers, we have deleted all previous mentions about the standardisation of the data from the manuscript.*

*Regarding 2021 as a valid counterfactual period. We use 2021 as the counterfactual since comparing happiness levels to a year such as 2019 would not make logical sense given that COVID-19 has had worldwide impacts changing people's social behaviour, thinking and response for the foreseeable future. The world can never be again as it was in 2019 (or any other plausible year before COVID-19) because COVID-19 is now a permanent part of our lives, with the prospect of another 'wave' of infections always on the horizon. Furthermore, following the parallel assumption of the DiD, we can see that 2021 is indeed a good counterfactual to use.*

*Additionally, as you would expect, there is a structural break in the data between before and after the first recorded cases of COVID-19 and, with time, the realisation of a worldwide pandemic.*

*Considering all these factors, we believe the days included in the analysis in the year 2021 are a suitable counterfactual since it represents 'new' happiness living with COVID-19.*

**3.3	I found some of the analysis difficult to follow. It seems that the authors have high-frequency (daily) data but much of the dif-in-dif is presented as if the unit of analysis is the year. This does not do justice to the fine time-varying data they have and could prove misleading if there is systematic measurement error that this level of aggregation introduces.**

*We thank the reviewer for this comment. In our analysis, we use high-frequency data. The year is only mentioned to identify the difference between the year of the event and the counterfactual. This is explained in more detail in section 3.*

**3.4	As Mark Bryan and Stephen Jenkins pointed out in their seminal paper if one aggregates data to the macro (country) level the analyst has to recognise that the N observations is countries not individuals. This has implications for the adjustment to standard errors. In this case, if I understand the dif-in-dif modelling, the data are micro level with country fixed effects, but of course individuals' responses are clustered within country. I think the authors need to spend more time explaining how they treat these data for the purposes of standard error adjustments since it may be that the significance of results depends upon this.**

*We thank the reviewer for this comment. Following point # 1.1, we highlight that we do not analyse individual tweets (we extract more than a million tweets daily with a vast number of tweets per country per day). Therefore, the unit of analysis is at the country-level. We do not cluster by country as we only have ten countries (rule of thumb states more than 40) and include only two years in the DiD analysis (the year of the event and the counterfactual). Hence, there is no need to control for correlation across panels.*

*We report robust standard errors to address heteroscedasticity.*

**3.5**	**Recent work shows just how sensitive some measures of wellbeing are to seasonality. One of these papers is in Plos One (Blanchflower, D. G. and Bryson, A. (2022)" COVID and Mental Health in America", PLoS ONE, 17(7): e0269855). It may be worth thinking more about this issue when trying to compare wellbeing across years. It may be that incorporating day/week controls is sufficient but it may be good to double-check.**

*We extend our sincerest thanks to the reviewer for pointing out this error in our empirical estimations. Subsequently, all models (DiD and event study) were re-estimated, and we now include a vector of control variables, including dummies for season (which includes the first two seasons in a year). Please see section 3.4.*

**4.	Data**

**4.1	I am not familiar with the GNH metric they construct and, perhaps like many other readers, are disinclined to hunt down other papers to find out. There should be a fulsome data appendix explaining precisely how the data are constructed – including the GNH – which also explains why the metric is appropriate, so that readers can check it out.**

**The GNH may, in fact, not be the best wellbeing metric since the growing literature on the measurement of wellbeing points to important differences in the determinants of and trends in positive and negative affect. They are asymmetrical in their responses to events such as those in this paper. If one had used a measure of negative affect we might have seen quite a different story. It would be a very nice addition if the authors could add in alternative wellbeing metrics to see whether negative affect responds differently to positive affect.**

*We thank the reviewer for this comment. Subsequently, we extensively rewrote section 3.2, explaining the construction of the GNH. Additionally, we state that the GNH is the net effect, considering both the positive and negative affect.*

*To support the GNH as a measure that captures well-being, we added a section on the external convergent validity of the GNH in the S2 Appendix. However, we also provide a brief paragraph below.*

*In essence, we assess the* *external convergent validity by comparing cross-sectional country rankings produced by the GNH to those available from alternative measures of well-being, such as the Eurobarometer's life satisfaction and the World Happiness Report (Helliwell et al., 2021). This exercise provided encouraging results: GNH-based country rankings are not significantly different from those based on alternative indicators.*

*References*

*Helliwell, J. F., Layard, R., Sachs, J., De Neve, J. E. (editors). (2021). World Happiness Report. Sustainable Development Solutions Network, New York.*

*Regarding the internal validity of the GNH, we also provide the reviewer with the following comprehensive discussion (also added as S1 Appendix of the revised manuscript).* ***Please note that this was part of ongoing research and development and includes information previously published.*** *Correlation between VADER and Sentiment140 is found in the manuscript on page 13.*

*We trust this will complement the convergent validity of the GNH to external data that represent the same or similar concept.*

***Internal validity of the GNH***

*To ensure the robustness of the GNH index, we conducted several tests, which included tweet volume. We report on these below:*

***Using four other dictionary-based sentiment classifiers*** *provided in the syuzhet package in R to test if the derived index portrays similar trends:*

- *Bing: A sentiment lexicon developed by Bing Lui and collaborators* [*https://www.cs.uic.edu/~liub/FBS/sentiment-analysis.html*](https://www.cs.uic.edu/~liub/FBS/sentiment-analysis.html)
- *AFINN: An evaluation of a word list for sentiment analysis in microblogs by Finn Årup Nielsen.*
- *NRC Word-Emotion Association Lexicon: A crowdsourced lexicon by Saif Mohammad* [*https://saifmohammad.com/WebPages/NRC-Emotion-Lexicon.htm*](https://saifmohammad.com/WebPages/NRC-Emotion-Lexicon.htm)
- *Syuzhet: Developed in the Nebraska Literary Lab under the direction of Matthew L. Jockers*

*Using these different lexicons for the GNH calculation yields the following graph.*

*As can be seen, the data is highly correlated, albeit biased. The Pearson correlation coefficients further justify this.*

*Ek moet vader add^^^*

```
##              sentiment140      bing     afinn       nrc   syuzhet
## sentiment140    1.0000000 0.8838716 0.8984890 0.8092620 0.8736781
## bing            0.8838716 1.0000000 0.9815833 0.9597444 0.9597691
## afinn           0.8984890 0.9815833 1.0000000 0.9347292 0.9608377
## nrc             0.8092620 0.9597444 0.9347292 1.0000000 0.9418306
## syuzhet         0.8736781 0.9597691 0.9608377 0.9418306 1.0000000
```

*Of note is that Pearson correlation coefficients lose meaning when the assumption of independence is not true. The correlation between one-step differences is also calculated to address this issue. Assuming independent increments, one sees that the data remain highly correlated.*

```
##              sentiment140      bing     afinn       nrc   syuzhet
## sentiment140    1.0000000 0.8713031 0.8700980 0.7937943 0.8669507
## bing            0.8713031 1.0000000 0.9742493 0.9553520 0.9747039
## afinn           0.8700980 0.9742493 1.0000000 0.9099610 0.9514972
## nrc             0.7937943 0.9553520 0.9099610 1.0000000 0.9500237
## syuzhet         0.8669507 0.9747039 0.9514972 0.9500237 1.0000000
```

*(Note that for 30 observations to be significantly correlated on a 0.01 level, one requires a Pearson correlation coefficient of around 0.463. The correlation coefficients obtained are well above this value. This, of course, is assuming all the standard statistical assumptions.)*

***Using timescale invariance*** *to determine whether the sampling period significantly influences the GNH values. Tweets are sampled per hour, the GNH is calculated, and then we use the daily average. What would happen, for example, if tweets were sampled daily or 6-hourly? Would the same measure be obtained?*

*The following compares the Daily GNH, and the 24h left rolling mean of the Hourly GNH. As can be seen, the data are very nearly the same. Indicating that the timescale at which sampling takes place does not influence the information obtained.*

***Using volume invariance*** *since the index is built from a sample of all the tweets with accounts with geolocation tagging enabled. To test if the volume directly influences the index, we sample 5k, 10k, 20k, and 30k tweets from the data every day.*

*As can be seen, the measure is robust against sampling. Even if we were to sample around 10% of the extracted tweets, there would still be a significant correlation with the measured GNH. This is justified once again with the following Pearson correlation coefficients:*

```
##           daily_gnh  Daily_5k Daily_10k Daily_20k Daily_30k
## daily_gnh 1.0000000 0.8766072 0.9171853 0.9803700 0.9902762
## Daily_5k  0.8766072 1.0000000 0.7908012 0.8523327 0.8590382
## Daily_10k 0.9171853 0.7908012 1.0000000 0.9023725 0.9169975
## Daily_20k 0.9803700 0.8523327 0.9023725 1.0000000 0.9707346
## Daily_30k 0.9902762 0.8590382 0.9169975 0.9707346 1.0000000
```

*Also, the one-step difference correlation coefficients:*

```
##           daily_gnh  Daily_5k Daily_10k Daily_20k Daily_30k
## daily_gnh 1.0000000 0.8157912 0.9130010 0.9678655 0.9869072
## Daily_5k  0.8157912 1.0000000 0.7321367 0.8081286 0.7926233
## Daily_10k 0.9130010 0.7321367 1.0000000 0.8609138 0.8913573
## Daily_20k 0.9678655 0.8081286 0.8609138 1.0000000 0.9377868
## Daily_30k 0.9869072 0.7926233 0.8913573 0.9377868 1.0000000
```

*(As noted previously, for 30 data points to be significantly correlated on a 0.01 level, the correlation coefficients need to exceed 0.463.)*

*Thus, taking a sample of the data does not significantly influence the metric.*

*Additionally, we provide you with the following evidence suggesting that the GNH captures a nation's mood. Please note that we did not include these explicitly in the revised manuscript. However, the reference to where readers could find country examples of the GNH capturing the mood of a nation has been included in section 3.2.*

***GNH captures the mood of a nation***

*Regarding what the GNH truly reflects, we offer the below country examples.*

*To consider the mood of a nation during a sporting event, the example of the South African Springboks winning the Rugby World Cup on 2 November 2019 was used. The rugby game commenced at 11:00 am (GMT+2) and lasted until 1:00 pm. The figure below shows the intraday per hour measurement of happiness levels. As can be seen, since waking, South Africans' happiness levels were higher than the norm of 6.3, caused by the higher expectations expressed for the day. At 11:00 am, there was a slight dip, but as the game proceeded and it became clear that the Springboks had the upper hand over their opponents, England, the happiness score increased to reach an hourly high of 7.9 at 1:00 pm (the end of the game). Coincidentally, this happiness level of 7.9 is the highest in South Africa since the index was launched in April 2019. This illustrates the power of sporting events to influence the mood of a whole nation.*

Happiness Index

| Hours | World Cup Saturday | Average Saturday |
| --- | --- | --- |
| 8 | 7.06147 | 6.8267 |
| 9 | 7.13236 | 6.578 |
| 10 | 7.18706 | 6.56248 |
| 11 | 6.69271 | 6.45993 |
| 12 | 7.23684 | 6.46694 |
| 13 | 7.94811 | 6.40578 |
| 14 | 7.57576 | 6.54192 |
| 15 | 6.37192 | 6.39388 |
| 16 | 6.46865 | 6.33812 |
| 17 | 6.32491 | 6.20428 |
| 18 | 6.80673 | 6.24551 |

*Source: Rossouw and Greyling (2020).*

*A political event also affects the mood of a nation; for example, in Australia, the federal elections were held on 18 May 2019. The figure below shows the happiness levels of Australians before and after the election day (18 May 2019). In the period leading up to the elections, the country's general mood was higher than the norm of 7.3. However, the happiness index showed a significant drop in the mood from 18 to 19 May 2019, as initially polls and TV channels reported that results favoured the Labor party. Still, then the results turned, and the Coalition party took the lead to win the elections against expectations. These events show that the happiness index reveals the mood of a nation during a political event.*

Happiness score

|  | Happiness score |
| --- | --- |
| 2019-05-15 | 7.3 |
| 2019-05-16 | 7.2958 |
| 2019-05-17 | 7.38 |
| 2019-05-18 | 7.377 |
| 2019-05-19 | 6.8416 |
| 2019-05-20 | 6.9799 |
| 2019-05-21 | 7.15 |
| 2019-05-22 | 7.31 |
| 2019-05-23 | 7.37 |

*Source: Rossouw and Greyling (2020).*

*Reference*

*Rossouw, S., & Greyling, T. (2020). Big Data and Happiness. In: Zimmermann, K.F. (eds) Handbook of Labor, Human Resources and Population Economics. Springer, Cham.* [*https://doi.org/10.1007/978-3-319-57365-6_183-1*](https://doi.org/10.1007/978-3-319-57365-6_183-1%20)

**5.**	**Heterogeneity**

**5.1	The literature on shocks indicates that their impact on wellbeing depletes with distance from the shock in both time and geography. I imagine this is what prompted the North v South investigation in the paper, although I couldn't see that clearly stated. I was wondering if there was a better way to capture distance from the shock by interacting that distance with the post-period? I didn't find the North v South analysis particularly illuminating.**

*We thank the reviewer for this comment. Subsequently, in the rewritten manuscript, we ensure that readers know that the inclusion of the Northern vs Southern hemisphere is not only to analyse the results in the different hemispheres but also as a robustness test of our findings for the whole sample. In the paper, we mention that proximity and spatial distribution play roles in determining the effect of a disaster on happiness. However, when it comes to our estimations, we keep all variables in the analysis the same to facilitate comparability across the whole sample and hemispheres.*

**6.	Magnitude of effects and the policy implications**

**6.1	The authors point to a very short-run dip in wellbeing after lockdown/Ukraine, with something that looks like regression to the mean shortly afterwards. This finding is common in the literature. The authors don't quantify the effect: just how big was it while it lasted? If it is not that big, and does not last that long, then why should we be concerned about it? This sort of question prompted Angus Deaton in a 2010 Oxford Economics Paper to wonder just how much policy makers needed to pay attention to such effects.**

**Somewhere one expects discussion of the magnitude of COVID (not lockdown) and Ukraine shocks to wellbeing if this paper is going to make a real contribution.**

*We thank the reviewer for raising this issue. Subsequently, we included the magnitude of the shocks (effect size), similar to Clark et al. (2020), in section 4.2.*

*While it is true that our event study confirms that adaptation to happiness levels seen in the counterfactual year occurred relatively quickly, it does not negate the negative spillover effects these macro-level shocks have in a relatively short period. In reaction to Russia's invasion of Ukraine, 4,888 people were detained during protests in 69 cities by 7 March 2022, most ending in violent clashes with authorities. OVD-Info (2022) reported at least 34 cases in which the police beat protesters on March 6. Additionally, there was a call for violence against not only Russian soldiers but also Russian civilians, which was allowed on Facebook (Reuters, 2022). Regarding the COVID-19 lockdown, we also note that, for example, South Africa experienced an average of eight protests per day in July 2020, the highest in a single month since 2013. These protests were driven in large by the experience of the first lockdown in March 2020, which coincided with allegations of corruption and mismanagement of funds linked to the fight against COVID-19 (Mengnjo, 2021).*

*References*

*Brodeur, A., Clark, A. E., Fleche, S., & Powdthavee, N. (2021). COVID-19, lockdowns and well-being: Evidence from Google Trends. Journal of Public Economics, 193:* *104346.*

*Clark, A. E., Doyle, O., & Stancanelli, E. (2020). The Impact of Terrorism on Individual Well-Being: Evidence from the Boston Marathon Bombing. The Economic Journal, 130(631), 2065–2104.*

*Mengnjo, T. T. (2021). Protests during the 2020 COVID-19 lockdown in South Africa. MoLab Inventory of Mobilities and Socioeconomic Changes. Department 'Anthropology of Economic Experimentation'. Halle/Saale: Max Planck Institute for Social Anthropology. Available from* [*https://www.eth.mpg.de/molab-inventory/securitizing-mobilities/protests-during-the-2020-covid19-lockdown-in-south-africa*](https://www.eth.mpg.de/molab-inventory/securitizing-mobilities/protests-during-the-2020-covid19-lockdown-in-south-africa)

*OVD-Info. (2022). Russia: Brutal Arrests and Torture, Ill-Treatment of Anti-War Protesters. Available from* [*https://www.hrw.org/news/2022/03/09/russia-brutal-arrests-and-torture-ill-treatment-anti-war-protesters*](https://www.hrw.org/news/2022/03/09/russia-brutal-arrests-and-torture-ill-treatment-anti-war-protesters)

*Reuters. (2022).* *Facebook allows war posts urging violence against Russian invaders. Available from* [*https://www.reuters.com/world/europe/exclusive-facebook-instagram-temporarily-allow-calls-violence-against-russians-2022-03-10/*](https://www.reuters.com/world/europe/exclusive-facebook-instagram-temporarily-allow-calls-violence-against-russians-2022-03-10/)

**7.	Minor points**

**7.1	the abstract is overly long and not very informative**

*We thank the reviewer for this comment. Subsequently, we have streamlined the abstract.*

**7.2	p. 10: I have no idea what NRC and VADER are.**

*We have extended these under the data section.*

**7.3	the figures aren't easy to read. It would be worth identifying t=0 and before/after periods on the x-axis**

*We thank the reviewer for this comment. After an extended empirical exercise, we have added new figures to the manuscript.*

**THE END**
