# Supplementary material for: Reactions to macro-level shocks and re-examination of adaptation theory using Big Data
Source: PLoS One. 2024 Jan 31;19(1):e0295896. doi: 10.1371/journal.pone.0295896 (PMC10830054; doi:10.1371/journal.pone.0295896)
Supplement: Attachment — Submitted filename: Comments on PONE-D-22-19460r1.docx [file pone.0295896.s006.docx]

**Comments on PONE-D-22-19460r1 “Re-examining adaptation theory using Big Data: Reactions to External Shocks”**

The revised paper is clearer than the original in terms of the nature of the shocks it is investigating, the data available and the nature of the analysis. But a number of issues remain which mean the paper is not ready for publication.

1. Macro analysis

Thanks for clarifying the point regarding the macro nature of the data which tracks country-level sentiment and thus provides the basis for cross-country comparison.

The checks on external validity are welcome because I think some will be sceptical about your ability to capture wellbeing for a representative population with twitter sentiment.

2. The shocks

In the revised paper the authors say they are testing responses to two shocks: a war and a ‘government regulation’ shock. The latter isn’t really the shock: it’s a policy response to the COVID shock. This is acknowledged but it’s tricky disentangling the two in that COVID is about the health shock (infection, illness, death) and the government response is lockdown. You control for COVID cases on the right-hand side of the equation. I’m really not sure that’s the right way to go since lockdown is, arguably, jointly determined with COVID cases, notwithstanding some cross-country variance in the policy.

3. Dif in Dif Estimation

I note the authors have consulted the new dif-in-dif literature I referred to on variance in the timing of events for the dif-in-dif across units. This is relevant to the depiction of before and after lockdown since this varies by country by a few weeks. As I said before, this variance can be important, especially for more granular analysis as in the event study, because the new dif-in-dif literature spends a lot of time discussing how to deal with potential confounding arising from combining different pre- and post-periods in pooled data when the timing of the ‘treatment’ differs across units. I can not see how you have accommodated these issues in your analysis and you do not clarify this in your response to reviewers.

I asked for a rendition of whether the dif-in-dif estimates are credible given their identification assumptions, eg. in relation to common trends. However, I didn’t see this addressed in the revision. And the issue of anticipation remains problematic.

4. A counterfactual for lockdown war

Previously I said it was hard to think of 2021 as a ‘counterfactual’ period given the dynamic and on-going effects of COVID. The authors said they had to have a post-COVID period for the counterfactual since COVID created a new ‘normal’. The problem is that it occurs after lockdown and not before, and it was not a stable new normal. So this is potentially really problematic. I can think of no other dif-in-dif estimation that that takes a post-period as the counterfactual.

5. The N observations

The fact that the data are aggregated to country level means we have a country-level analysis. As such the N is low and the authors need to consider the implications of this for their analysis. I suggest the authors go back to the Bryan and Jenkins paper here <https://academic.oup.com/esr/article/32/1/3/2404247> to establish whether their results may be unreliable for the reasons discussed.

6. Heterogeneity

I remain unclear as to the value of the North v South analysis. And the paper does not deal with the issue I raised last time, namely the idea that the impact of shocks on wellbeing depletes with distance from the shock in both time and geography. It would be good to see this with respect to Ukraine.

7. Habituation and effect size

The current estimates suggest the size of the wellbeing shock was bigger for lockdown that Ukraine but that the Ukraine effect lasted longer. Why would this be? You also suggest that the Ukraine effect lasted four weeks. This is a long time in the wellbeing literature. I suggest you have regard to the terrorist literature I referred to in my previous comments. It’s worth thinking about these effects relative to what you find.
