# Supplementary material for: Reactions to macro-level shocks and re-examination of adaptation theory using Big Data
Source: PLoS One. 2024 Jan 31;19(1):e0295896. doi: 10.1371/journal.pone.0295896 (PMC10830054; doi:10.1371/journal.pone.0295896)
Supplement: Attachment — Submitted filename: Response to reviewer.docx [file pone.0295896.s007.docx]

SECOND RESPONSE TO REVIEWER #2

**Manuscript ID PONE-D-22-19460R1**

**Re-examining adaptation theory using Big Data: Reactions to external shocks.**

We thank reviewer #2 for the additional comments. Each has been comprehensively addressed, as shown below (*authors' responses shown in italics*). We believe the paper to be significantly improved due to the additional comments received from the reviewer.

**Reviewer #2's comments addressed by the authors.**

**The revised paper is clearer than the original in terms of the nature of the shocks it is investigating, the data available and the nature of the analysis. But a number of issues remain which mean the paper is not ready for publication.**

*We thank the reviewer for their generally positive view and trust that we have adequately addressed your remaining concerns.*

1. **Macro analysis**

**Thanks for clarifying the point regarding the macro nature of the data which tracks country-level sentiment and thus provides the basis for cross-country comparison.**

**The checks on external validity are welcome because I think some will be sceptical about your ability to capture wellbeing for a representative population with twitter sentiment.**

*We thank the reviewer for raising this point. We wish to highlight that we do not claim that the data is representative and specify this in section 3.2 of the manuscript: "Although we cannot assume that Twitter users are representative of a country, we know that a vast number of the population do have Twitter accounts, and the number of tweets approximates millions per day. Therefore, biases are limited by the size of the sample."*

2. **The shocks**

**In the revised paper the authors say they are testing responses to two shocks: a war and a 'government regulation' shock. The latter isn't really the shock: it's a policy response to the COVID shock. This is acknowledged but it's tricky disentangling the two in that COVID is about the health shock (infection, illness, death) and the government response is lockdown. You control for COVID cases on the right-hand side of the equation. I'm really not sure that's the right way to go since lockdown is, arguably, jointly determined with COVID cases, notwithstanding some cross-country variance in the policy.**

*We thank the reviewer for pointing out this nuanced relationship. The lockdown was indeed due to the COVID-19 health shock. Therefore it is intuitive to control for the health shock in the regression if we are interested in the government regulation shock. A similar shock could have occurred notwithstanding COVID-19, for example, after a nuclear disaster allowing minimal travel outside the home. The only way to measure the government regulation shock is to control for the coronavirus, thus removing the relationship from the disease itself and only considering that people were confined to their homes.*

3. **Dif in Dif Estimation**

**I note the authors have consulted the new dif-in-dif literature I referred to on variance in the timing of events for the dif-in-dif across units. This is relevant to the depiction of before and after lockdown since this varies by country by a few weeks. As I said before, this variance can be important, especially for more granular analysis as in the event study, because the new dif-in-dif literature spends a lot of time discussing how to deal with potential confounding arising from combining different pre- and post-periods in pooled data when the timing of the 'treatment' differs across units. I can not see how you have accommodated these issues in your analysis and you do not clarify this in your response to reviewers.**

**I asked for a rendition of whether the dif-in-dif estimates are credible given their identification assumptions, eg. in relation to common trends. However, I didn't see this addressed in the revision. And the issue of anticipation remains problematic.**

*Dear reviewer,*

*We have thoroughly read and worked through De Chaisemartin and d'Haultfoeuille (2020), Roth et al. (2022) and Callaway and Sant'Anna (2021). Additionally, we worked through all the STATA help files pertaining to this matter.*

*When considering Callaway and Sant'Anna’s (2021) study, you will notice that they compare different units (US counties) – where the treatment starts in different time periods (years). In the first period, there is a "never treated" group which is the comparison group. Next, they give the year in which each unit (US counties) first started the treatment (minimum wage). They assume that once a unit receives the treatment, they continuously receive it. They aggregate those counties where the treatment started in the same year, for example, 2006 or 2007. For the DiD, they compare the year in which the treatment was started to the control year of no treatment – which they assume initially is 2003, and thereafter to each of the following years – for example, t2003-2004 and t2004-2005. The units (counties) differ across the whole period. As mentioned, units starting the treatment in the same years are aggregated to get the necessary results.*

*Our data differs from the abovementioned. You are correct that the timing of the events during lockdown differs between countries. However, all the countries in our sample are treated in the year of the shock – year 1 – and a "no treatment" unit (country) doesn't exist. Furthermore, in year 2, none of our countries are treated.*

*In the paper by Callaway and Sant'Anna (2021), the assumption of the" Irreversibility of Treatment" is made. However, this assumption can not be applied to our data as it is a mean reverting process.*

*Furthermore, our units are not different across time 1 and time 2 but exact replicas. Therefore, we compare the same unit (country) at two different time periods (years). Our counterfactual or control group is the same country in time 2 (where no treatment was applied). Time 1 and time 2 include the exact same dates around the lockdown/invasion – thus, lockdown is 0 with ten days before and ten days thereafter. For example, New Zealand locked down on 23 March in time 1 – our counterfactual is the same period and day in time 2. A country treated in time 1 is compared to the same country not treated in time 2.*

*According to Callaway and Sant'Anna (2021), the question and the purpose of the analysis must be considered in deciding the appropriate estimation technique. The abovementioned technique does not give us the required results to address our research question. We follow the same method as other studies (please see Brodeur et al. 2021).*

*Below are the results obtained through STATA using Callaway and Sant'Anna's (2021) dataset retrieved from GitHub, where the relationship between employment and the introduction of minimum wages in different counties in the US was analysed. As mentioned before, the introduction of the minimum wage is the treatment. Different counties introduced it in different years.*

*It is important to note that the control group shifts to when the treatment occurs. For example, if the treatment only occurred in 2006 (g2006), the final control group used was the year before treatment, 2005. Therefore, when we consider the years t2005-2007, we notice that employment decreased by -.012 due to the implementation of the minimum wage.*

*If we apply the same coding to our data, we get output but with no estimations, since we do not have a single "no treatment unit" (country) as a control group. Therefore this method is not the appropriate one for our current study.*

*References:*

*Brodeur, A., Clark, A. E., Fleche, S., & Powdthavee, N. (2021). COVID-19, lockdowns and well-being: Evidence from Google Trends. Journal of Public Economics, 193: 104346.*

*Callaway, B., & Sant’Anna, P. H. (2021). Difference-in-Differences with multiple time periods. Journal of Econometrics, 225(2), 200-230.*

*De Chaisemartin, C., & d’Haultfoeuille, X. (2020). Two-Way Fixed Effects Estimators with Heterogeneous Treatment Effects. American Economic Review, 110(9), 2964-2996.*

*Roth, J., Sant' Anna, P. H. C., Bilinski, A., & Poe, J. (2022). [What's Trending in Difference-in-Differences? A Synthesis of the Recent Econometrics Literature](https://ideas.repec.org/p/arx/papers/2201.01194.html).* [*Papers*](https://ideas.repec.org/s/arx/papers.html) *2201.01194, arXiv.org, revised Jan 2023.*

*Regarding the identification strategy, we wish to highlight that we do specify in section 3.4.1 the following:*

*"The identification strategy in equation (1) relies on the assumption that, in the absence of lockdown and invasion, the GNH will have followed the same trends as in the counterfactual year, thus a common-trend assumption. However, we recognise that the trends in the years 2020/2022 and 2021 are not always in perfect unison, and therefore the strict assumption of parallel trends is not fully fulfilled. This is visible from Fig 1 and Fig 2 in section 4.1, where we see similar trends (not perfectly unison) in the year of the events and the counterfactual year prior to the shocks. Nonetheless, our identification assumptions are further supported as the dates of the shocks differ across countries; therefore, each* *country has a unique day 0 (the date of the announcement of the first lockdown). It follows then that if a similar reaction is noticed on the GNH across countries on the day of the announcement, it is most likely due to the shock.*

*Similarly, the invasion was a unique event, and none of the countries directly participated in the war. Therefore, it is most likely that the observed decrease in GNH across the countries in our sample on the day of the invasion is related to the shock. We also note from the narrative of the extracted tweets that after the lockdown and invasion dates, the tweets related to these events intensified comparatively to 2021, indicating that these events influenced the narrative of people and mattered for their happiness levels, strengthening our case for identification (please also refer to the work done by Brodeur et al. [54])."*

*When considering anticipation, we need to note the following. We ran our models using the announcement date of the lockdowns. Whereas the actual lockdown dates could capture a larger portion of the anticipatory effect, it is less so with the announcement dates, given that much uncertainty regarding possible non-pharmaceutical interventions was at the order of the day. Additionally, the exact form country's lockdown would take (localised vs national) and the exact timing of its onset (before the announcement) and ferocity were unanticipated and not foreseen by anybody. The same can be said for the greatest financial shock in recent memory (2008). Whereas many institutions warned about imbalances in financial markets and the global economy and anticipated the crisis, the extent of the financial shock could not be anticipated.*

4. **A counterfactual for lockdown war**

**Previously I said it was hard to think of 2021 as a 'counterfactual' period given the dynamic and on-going effects of COVID. The authors said they had to have a post-COVID period for the counterfactual since COVID created a new 'normal'. The problem is that it occurs after lockdown and not before, and it was not a stable new normal. So this is potentially really problematic. I can think of no other dif-in-dif estimation that that takes a post-period as the counterfactual.**

*We thank the reviewer for raising this point and allowing us to address this properly. As you know, the guiding idea behind counterfactual analyses of causation is the thought that – as David Lewis puts it – "We think of a cause as something that makes a difference, and the difference it makes must be a difference from what would have happened without it. Had it been absent, its effects – some of them, at least, and usually all – would have been absent as well" (1973b).*

*Like most contemporary counterfactual theories, Lewis's theory employs possible world semantics for counterfactuals. Such semantics states truth conditions for counterfactuals regarding similarity relations between possible worlds (Menzies & Beebee, 2020). Lewis famously advocates realism about possible worlds, according to which non-actual possible worlds are real concrete entities on a par with the actual world (Lewis, 1986). However, most contemporary philosophers would seek to deploy the explanatorily fruitful possible worlds framework while distancing themselves from full-blown realism about possible worlds.*

*It is important to remember that the central notion of possible world semantics for counterfactuals is a relation of comparative similarity between worlds (Lewis, 1973a). One world is said to be closer to actuality than another if the first resembles the actual world more than the second does.*

*According to Bennett (1987: 369), "If we are to counterfactualize about particulars, we must be able to distinguish worlds at which a given event does not occur at all from ones at which it occurs but is somewhat unlike the way it actually was."*

*Therefore, we maintain that it would not be appropriate to use 2019 as a counterfactual since it could not resemble the actual world of 2020 better than 2021 simply because the world can never again go back to 2019 and live without the lasting changes COVID-19 has had on economies, society and well-being.*

*We have read up extensively on this issue, and nowhere can we find that a counterfactual is required to "proceed with the event". More significantly, there is no statistical reason why we cannot use a counterfactual after the event. The only important issue is that our choice of the counterfactual world is closest to the actual world in question.*

*References:*

*Bennet, J. (1987). Event Causation: The Counterfactual Analysis. Philosophical Perspectives, 1, 367-386.*

*Lewis, D. (1973a). Counterfactuals, Oxford: Blackwell.*

*Lewis, D. (1973b). Causation. Journal of Philosophy, 70: 556–67.*

*Lewis, D. (1986). The Plurality of Worlds, Oxford: Blackwell.*

*Menzies, P., & Beebee, H. (2020). Counterfactual Theories of Causation. The Stanford Encyclopedia of Philosophy (Winter 2020 Edition), Edward N. Zalta (ed.).*

5. **The N observations**

**The fact that the data are aggregated to country level means we have a country-level analysis. As such the N is low and the authors need to consider the implications of this for their analysis. I suggest the authors go back to the Bryan and Jenkins paper here** [**https://academic.oup.com/esr/article/32/1/3/2404247**](https://academic.oup.com/esr/article/32/1/3/2404247) **to establish whether their results may be unreliable for the reasons discussed.**

*We thank the reviewer for this comment. Our N (countries) is indeed relatively small compared to our T (time periods). You referred us to the Bryan and Jenkins paper, which relates to multilevel modelling estimations, similar to the Fixed Effect estimations commonly used in econometrics. Therefore considering the paper, we tested for stationarity using the Im–Pesaran–Shin panel unit-root test (Im et al. 2003). We found the presence of a first-grade unit root. We subsequently introduced a lagged dependent variable (*${GNH}_{i-1,c}$*) in our model (Greene 2010) to attain stationarity. The above-discussed did not significantly change the results pertaining to our DiD estimator, although the DiD estimator is somewhat smaller.*

*References:*

*Greene, H. W. (2010). Econometric analysis. Chapter 11 in Models for panel data. Pearson Education Limited 2012.*

*Im, K., Pesaran, M., & Shin, Y. (2003). Testing for Unit Roots in Heterogeneous Panels. Journal of Econometrics, 115, 53–74.*

6. **Heterogeneity**

**I remain unclear as to the value of the North v South analysis. And the paper does not deal with the issue I raised last time, namely the idea that the impact of shocks on wellbeing depletes with distance from the shock in both time and geography. It would be good to see this with respect to Ukraine.**

*We thank the reviewer for this comment. Previously, we noted that we had rewritten the manuscript to ensure that readers know the inclusion of the Northern vs Southern hemisphere is not only to analyse the results in the different hemispheres but also as a robustness test of our findings for the whole sample (pages 4 and 27 of the manuscript). We also mention on pages 5 and 25 of the manuscript that proximity and spatial distribution play roles in determining the effect of a disaster on happiness.*

*Page 5 - "Regarding the Ukraine invasion, the Northern hemisphere has similar results as the analysis for all the countries. However, adaptation seems somewhat quicker for the Southern hemisphere, with GNH levels positive and significant by week two post-event. This is plausible since proximity and spatial distribution play vital roles in determining the effect of a disaster on happiness (Rehdanz et al., [14])."*

*Page 25 – "Regarding the invasion, we note that the Northern hemisphere has similar results as the analysis for all the countries. Even though not statistically significant, the GNH remained lower for three weeks post-event before adapting in week 4. However, adaptation seems somewhat quicker for the Southern hemisphere, with GNH levels positive and significant by week three post-event. This is plausible since proximity and spatial distribution play vital roles in determining the effect of a disaster on happiness (Rehdanz et al., [14])."*

*However, we maintain that when it comes to our estimations, we keep all variables in the analysis the same to facilitate comparability across the whole sample and hemispheres.*

7. **Habituation and effect size**

**The current estimates suggest the size of the wellbeing shock was bigger for lockdown that Ukraine but that the Ukraine effect lasted longer. Why would this be? You also suggest that the Ukraine effect lasted four weeks. This is a long time in the wellbeing literature. I suggest you have regard to the terrorist literature I referred to in my previous comments. It's worth thinking about these effects relative to what you find.**

*We thank the reviewer for this comment. In the manuscript, we pointed out that the magnitude of the lockdown shock was bigger than that of the invasion of Ukraine. However, this is expected as the lockdown directly affected all countries in the sample, whereas the invasion of Ukraine had an indirect effect. Furthermore, you will notice that in the re-estimation of all the models, adaptation in the invasion shock occurs in week three rather than week four.*

*We discuss this indirect effect further on page 24 – "* *Therefore, considering the adaptation period, we find that, even though the lockdown had a bigger effect on happiness than the invasion, the associated adaptation period is shorter for the former compared to the latter. After the lockdown shock, happiness adapted two weeks later. In contrast, the effects remained longer for the invasion shock and only adapted in the third week, with weeks one and two showing no significant differences from the event week. This result is plausible since the invasion created a ripple effect throughout the countries in our study. At first, the people were negatively affected by sadness and sympathy for Ukraine. Still, as time passed, the countries themselves were impacted by rising gas prices, inflation, etc. Therefore, the invasion turned an initial Ukraine problem into an all-country problem, and we only see in week three that the happiness levels were positive and significantly related to the event week (thus adapting to happiness levels before the event week)."*

*In terms of the length of effect on happiness, our estimations of two weeks (lockdown) and three weeks (Ukraine invasion) are in line with other research (both at a macro level and a micro level – please see our literature review, and in addition, please note the following:*

*In a study conducted by Metcalfe et al. (2011), they investigated the effects of 9⁄11 on the mental well-being of those living in the UK (a country not directly impacted by the terrorist attack).*

*For the year 2001, their analysis split their initial sample of 15,587 observations into two groups – the pre-9/11 subsample (or the control group), which consists of individuals who were surveyed between 1 January 2001 and 10 September 2001, and the post-9⁄11 subsample, which consists of individuals who were surveyed between 12 September 2001 and 31 December 2001.*

*To determine whether the effect of the 9⁄11 attacks heightened or dissipated in the weeks that followed the event, they split the post-9⁄11 group into four sub-samples: 12 September 2001–20 September 2001; 21 September 2001–30 September; 1 October 2001–31 October 2001; 1 November 2001–30 November 2001; and 1 December 2001–31 December 2001. They found a significant and negative impact until late September, with the additional possibility that the effect could have lingered as long as November.*

*Additionally, a study done by Ford et al. (2003) to investigate feelings, perceptions and behaviours of respondents interviewed within 9 weeks after the 9/11 attacks found that those respondents that reported higher levels of sadness returned to baseline in 4 to 6 weeks.*

*Lastly, Knudsen et al. (2005) investigated the effect of 9/11 on alcohol consumption and depressive feelings. They found that respondents interviewed within the first 2 weeks post-9/11 reported significantly more depressive symptoms in the previous week than their pre-9/11 counterparts, such that the expected count of depressive symptoms was 55.2 per cent greater in this first post-9/11 group. An elevated number of depressive symptoms continued to be reported among respondents interviewed during the following 2-week period. The expected count of depressive symptoms for respondents interviewed between 2 and 4 weeks was 21.2 per cent greater than the number of symptoms reported by pre-9/11 respondents. However, beginning with those respondents interviewed between 4 and 6 weeks and continuing through to the end of the study period, there were no further significant differences between the pre-9/11 and post-9/11 groups.*

*Therefore, given the above studies, we maintain that our estimate of an effect on happiness lasting up to three weeks for the invasion of Ukraine is acceptable, given the current literature and highly plausible.*

*References:*

*Ford, C. A., Richard J. U., Gleiter, K., & Chantala, K. (2003). Reactions of Young Adults to September 11, 2001. Archives of Pediatric and Adolescent Medicine, 157(6), 572e578.*

*Knudsen, H. K., Roman, P. M., Johnson, J. A., & Ducharme, L. J. (2005). A changed America? The effects of September 11th on depressive symptoms and alcohol consumption. Journal of Health and Social Behavior, 46(3),260-73.*

*Metcalfe, R., Powdthavee, N., & Dolan, P. (2011). Destruction and Distress: Using a Quasi-Experiment to Show the Effects of the September 11 Attacks on Mental Well-Being in the United Kingdom. The Economic Journal, 121(550), F81–F103.*

**THE END**
