# Supplementary material for: Reactions to macro-level shocks and re-examination of adaptation theory using Big Data
Source: PLoS One. 2024 Jan 31;19(1):e0295896. doi: 10.1371/journal.pone.0295896 (PMC10830054; doi:10.1371/journal.pone.0295896)
Supplement: Attachment — Submitted filename: Response to reviewer.docx [file pone.0295896.s009.docx]

THIRD RESPONSE TO REVIEWER #2

**Manuscript ID PONE-D-22-19460R2**

**Re-examining adaptation theory using Big Data: Reactions to external shocks.**

We thank reviewer #2 for the additional comments. We have answered your questions below (*authors' responses shown in italics*). However, we agree with you that we will have to agree to disagree on most points and that the editor should now decide how to handle these differences.

**Reviewer #2's comments.**

**The authors have responded in detail to my previous points and have put considerable effort into the paper.**

*We thank the reviewer for this comment.*

**I remain concerned about a couple of points where, I suspect, we will have to agree to disagree. It will be for the editor to decide how to handle those differences.**

*We agree wholeheartedly with the reviewer.*

**Points raised:**

1. **The first is the issue of treating lockdown as a 'shock' since it is really COVID that was the shock and the lockdown was a response to that shock. I appreciate that the paper is not the first to do this. Even so, I find it hard to interpret the impact of lockdown with COVID used as independent right-hand side variables.**

*As the reviewer states, we are not the first study that proved that lockdown is a shock impacting well-being. Here, you only need to consider the number of people who became unemployed or could not earn an income (entrepreneurs), lower consumption levels or the negative impact on social relationships.*

*We are also not the first study to use COVID-19-related variables as independent covariates. Our study aligns with prior scholarly inquiries wherein lockdown measures have been utilised as an exogenous perturbation affecting well-being. Additionally, incorporating COVID-19-related variables as independent covariates has been a recurrent practice in the literature. This methodological approach is imperative, given the intricacies associated with isolating the unique impact of lockdown on well-being. A failure to control for the effects of COVID-19 could undermine the validity of attributing observed effects solely to lockdown measures rather than the broader influence of the COVID-19 pandemic itself.*

*For your reference, please see the studies below that investigated the effect of lockdown or used COVID-19-related variables as independent covariates.*

*Studies investigating lockdown*

- *Ohrnberger (2022) investigated the causal effect of the lockdown shock on health.*

*Ohrnberger, J. (2022). Economic shocks, health, and social protection: The effect of COVID-19 income shocks on health and mitigation through cash transfers in South Africa. Health Economics, 31(11), 2481-2498.*

- *Mejean (2022) discusses at length how the lockdown in early 2020 shocked the business world.*

*Mejean, I. (2022). The lockdown supply shock. Available from* [*https://cepr.org/multimedia/lockdown-supply-shock*](https://cepr.org/multimedia/lockdown-supply-shock)

- *Blanchflower and Bryson (2022) discussed how the US's economic shock occurred in response to policy efforts to contain the COVID-19 virus by going into lockdown in March 2020.*

*Blanchflower, D.G., & Bryson, A. (2022). Covid and mental health in America. PLoS ONE 17(7):e0269855*

- *Adams-Prassl et al. (2022) compare mental health before and after the introduction by state of lockdowns.* *They find that "the negative impact of the lockdown orders is entirely driven by a negative impact on women".*

*Adams-Prassl A., Boneva T., Golin M, and Rauh C. (2022). The impact of the coronavirus lockdown on mental health: evidence from the United States, Economic Policy, eiac002.*

- *Hamermesh (2020) examined data from the American Time Use Survey for 2012–2013 and used those data to simulate what would happen in lockdowns, given that this would result in people staying at home. He predicted a rise in life satisfaction for married and a decline for singles due to loneliness due to more time spent alone.*

*Hamermesh D. S., Life satisfaction, loneliness and togetherness, with an application to Covid-19 lockdowns, Review of Economics of the Household, 18: 983–1000 (2020). https://doi.org/10.1007/s11150-020-09495-x PMID: 32837455*

- *Brodeur et al. (2021)* *used Google Trends data to test whether the lockdowns implemented in Europe and America led to changes in well-being-related topic search terms. Their results suggest that the lockdown may have severely affected people's mental health.*

*Brodeur, A., Clark A., Fleche S., and Powdthavee N., COVID-19, lockdowns and well-being: Evidence from Google trends,' Journal of Public Economics, 193, 104346 (2021).*

- *Mahmud and Riley (2021) provided evidence of the economic and well-being impact of the COVID-19 lockdown shock on a sample of households in rural Uganda. Their results were among the first to show a large negative impact of the lockdown on a rural population.*

*Mahmud, M., & Riley, E. (2021). Household response to an extreme shock: Evidence on the immediate impact of the Covid-19 lockdown on economic outcomes and well-being in rural Uganda. World Development, 140, 105318.*

- *Sibley et al. [31] documented increased anxiety/depression following lockdown and warned about long-term challenges to mental health in New Zealand.*

*Sibley, C.G., Greaves, L., Satherley, N., Wilson, M., Lee, C., Milojev, P., et al. (2020). Short-term Effects of the COVID-19 Pandemic and a Nationwide Lockdown on Institutional Trust, Attitudes to Government, Health and Wellbeing. APA PsycNet, 75(5): 618–63.*

*Studies using COVID-19-related variables as independent covariates.*

- *Rossouw et al. (2021) used the number of COVID-19 cases and the squared number of COVID-19 cases as independent covariates.*

*Rossouw, S., Greyling, T. & Adhikari, T. (2021). Happiness-lost: Did Governments make the right decisions to combat Covid-19?* *South African Journal of Economic and Management Sciences, 24(1), a3795.*

- *Brodeur et al. (2021)* *used lagged number of new deaths of COVID-19 per day per million in a country or State as an independent covariate.*

*Brodeur, A., Clark A., Fleche S., and Powdthavee N., COVID-19, lockdowns and well-being: Evidence from Google trends,' Journal of Public Economics, 193, 104346 (2021).*

- *Greyling et al. (2021) used new COVID-19 deaths as an independent covariate.*

*Greyling, T., Rossouw, S. & Adhikari, T. (2021). The good, the bad and the ugly of lockdowns during Covid-19. PLOS ONE, 16(1), e0245546.*

- *Sarracino et al. (2023) used daily new COVID-19 cases as an independent covariate.*

*Sarracino F, Greyling T, O'Connor K, Peroni C, Rossouw S (2023) A year of pandemic: Levels, changes and validity of well-being data from Twitter. Evidence from ten countries. PLoS ONE 18(2): e0275028.*

- *Rossouw et al. (2021) used the number of COVID-19 cases as an independent covariate.*

*Rossouw S, Greyling T, Adhikari T (2021) The evolution of happiness pre and peri-COVID-19: A Markov Switching Dynamic Regression Model. PLoS ONE 16(12): e0259579*

- *Greyling et al. (2021) used the number of new COVID-19 deaths as an independent covariate.*

*Greyling, T., Rossouw, S. & Adhikari, T. (2021). A tale of three countries: How did Covid-19 lockdown impact happiness? South African Journal of Economics, 89(1), 25-43.*

2. **Second, I think that treating 2021 as a counterfactual period is problematic due to the dynamic and on-going effects of COVID. It's very tricky interpreting results relative to a counterfactual period which is itself unstable.**

*The reviewer notes that COVID-19 is the shock, but as we explained and shown in point #1, many previous studies (within PLOS ONE itself) and our own study's results unequivocally show that lockdown was a negative shock on well-being. Therefore, considering that the first lockdown is the year of the shock (2020), it should be compared to a very similar period except for the shock (2021).*

*In the previous response, we quoted from the works of experts regarding counterfactuals* *(Bennet, 1987; Lewis, 1973a and b, 1986; Menzies & Beebee, 2020),* *and it was extremely clear that when considering a counterfactual, it should be closer to actuality than another if the first resembles the actual world more than the second does.* *One simply cannot state that 2019 is a better counterfactual than 2021 because 2019 is not closer to actuality in 2020. There was no COVID-19 in 2019. There was COVID-19 and lockdown in 2020, and only COVID-19 in 2021. How can one say that 2019 is closer to 2020 in actuality than 2021?*

*If it is not the actuality that you question but the fact that we use a counterfactual which follows the shock, we offer the following:*

*Menzies and Beebee (2020) and Lewis (1973a) state, "Counterfactuals play a central role in the new theory, as in the old. However, the counterfactuals it employs do not simply state the dependences of whether one event occurs or whether another event occurs.* *The counterfactuals state dependences of whether, when, and how one event occurs on whether, when, and how another event occurs. A key idea in the formulation of these counterfactuals is that of an alteration of an event. This is an actualised or unactualised event that occurs at a slightly different time or in a slightly different manner from the given event. An alteration is, by definition, a very fragile event that could not occur at a different time or in a different manner without being a different event. Lewis intends the terminology to be neutral on the issue of whether an alteration of an event is a version of the same event or a numerically different event".*

*Therefore, you will notice from the above there is no requirement for the time period of the event, i.e., implying that the year 2021 cannot serve as a counterfactual for 2020. The only requirement is that "This is an actualised or unactualised event that occurs at a slightly different time".*

*Ultimately, we maintain that it would not be appropriate to use* *2019 as a counterfactual since it could not resemble the actual world of 2020 better than 2021 simply because the world can never again go back to 2019 and live without COVID-19.* *Additionally, 2021 has already absorbed the effects of COVID-19, and what we see in the graphs and from our econometric analysis is the shock on happiness due to the lockdown, not the ongoing effects of COVID-19. Subsequently, we added a discussion on the chosen counterfactual under section 3.1 on page 11.*

*References:*

*Bennet, J. (1987). Event Causation: The Counterfactual Analysis. Philosophical Perspectives, 1, 367-386.*

*Lewis, D. (1973a). Counterfactuals, Oxford: Blackwell.*

*Lewis, D. (1973b). Causation. Journal of Philosophy, 70: 556–67.*

*Lewis, D. (1986). The Plurality of Worlds, Oxford: Blackwell.*

*Menzies, P., & Beebee, H. (2020). Counterfactual Theories of Causation. The Stanford Encyclopedia of Philosophy (Winter 2020 Edition), Edward N. Zalta (ed.).*

3. **Third, I'd like to have seen some engagement with the common trends assumption in the paper but there is none despite the fact that it's crucial to dif-in-dif estimation. If there is no common trend, then one needs to resort to something to tackle that such as synthetic matched dif-in-dif.**

*We note the reviewer's concern, and, therefore, we engage in a more in-depth discussion regarding our assumption of parallel trends in the manuscript.*

*Our understanding is as follows: the assumption is about what would happen in the future had treatment not occurred, which is not something we can test. However, we can observe whether the treated (the year of the shock 2020/2022) and control (the counterfactual year 2021) have parallel trends. If this is true, it is more credible that they might have followed parallel trends for more periods had treatment (the shocks) not occurred. Using time-series data implies that we must have common trends of two time periods rather than comparing similar individuals or units. This has its challenges, as two time periods will seldom have the exact same trend.*

*We can see this challenge in other papers that used DiD comparing time-series data. For example, when you look at the study published by Brodeur, A., Clark, A., Fleche, S., and Powdthavee, N. (2021). COVID-19, lockdowns and well-being: Evidence from Google trends. Journal of Public Economics, 193, 104346; you will see from their graphs below that there are common trends, but they are not perfect.*

*Furthermore, we are familiar with the developments over the last years on different methods to address the problem if the assumption of parallel trends is not precisely met. The following papers give an overview of these new trends:*

- *Clément de Chaisemartin & Xavier D’Haultfœuille, 2023. "Two-way fixed effects and differences-in-differences with heterogeneous treatment effects: a survey****,****" The Econometrics Journal, vol 26(3), pages C1-C30.*
- *Roth, J., Sant' Anna, P. H. C., Bilinski, A., & Poe, J. (2022). What's Trending in Difference-in-Differences? A Synthesis of the Recent Econometrics Literature. Papers 2201.01194, arXiv.org, revised Jan 2023.*
- *Rambachan, A., & Roth, J. (2022). A More Credible Approach to Parallel Trends. Conference proceedings. Available from* [*https://www.semanticscholar.org/paper/A-More-Credible-Approach-to-Parallel-Trends-%E2%88%97-Rambachan-Roth/fe8faa36f6fecbb09d255a06b3bd8af2ecce0e41*](https://www.semanticscholar.org/paper/A-More-Credible-Approach-to-Parallel-Trends-%E2%88%97-Rambachan-Roth/fe8faa36f6fecbb09d255a06b3bd8af2ecce0e41)

*However, these methods are specific to matched individuals or units, not time-series data. We also discussed this phenomenon in the previous response and tested the suggested method by Callaway, B., & Sant'Anna, P. H. (2021). Difference-in-Differences with multiple time periods. Journal of Econometrics, 225(2), 200-230. Therefore, it is unlikely to find a perfect common trend when considering a common trend in time-series data.*

*However, to strengthen the assumption, we use context-specific knowledge to argue why we selected the specific counterfactual time period related to the years in which shocks occurred and, thus, why parallel trends are more credible.*

*Our selection of the time periods, 2021 and 2020 and 2021 and 2022, respectively, had the COVID-19 pandemic in common since it occurred across all the selected time periods. Furthermore, as the time periods considering both shocks correspond exactly to the time periods of the counterfactual, we also find other similarities. These similarities include i) the seasons are similar, and ii) many public holidays in the countries are on the same date in 2020 and 2021, and the same for 2021 and 2022 –* *often capturing higher happiness levels on these days. We also considered other factors that hold both in the year of the counterfactual and the years of the shock, for example, higher unemployment rates, slower GDP growth and decreased consumer spending. These factors once again strengthen our assumption of common trends. In 2022, we saw a recovery, but as we analyse February 2022, the recovery was not as evident as by the end of the year.*

*Nonetheless, we recognise that we do not have perfect parallel trends – and the main reason for this assumption is to prove causality – i.e., the shocks caused the decrease in happiness. As this assumption does not hold perfectly, we interpret the results cautiously and claim that there is a* *strong relationship between the shocks and the decrease in happiness.*

*To test the robustness of our results in the DiD estimation, we employ event studies, which clearly point out the causality of events—showing that in the week after the shocks, happiness was lower than before.*

*Considering the results of these different estimation techniques jointly, we are confident that the shocks led to lower happiness levels.*

*Subsequently, we added a discussion in section 3.4.1 (pages 17 and 18) to explain the reasons that the assumption of common trends most likely holds in the lockdown and the invasion of Ukraine shocks. However, the causality should be interpreted cautiously, and we warn about interpreting the results on page 18, line 457 and page 19, line 495.*

*Furthermore, we plot graphs with an extended time period before the shocks (we added four more days). You will notice that though the trends are not perfectly parallel, the trends before the lockdown and invasion show they are closely related. In addition, after some lapse of time after the shocks, we seem to find parallel trends, thus strengthening our assumption. Please see below.*

4. **Fourth, I still don't see the value of the North v South analysis. If it's intended to capture intensity of treatment it's a pretty crude way of doing so.**

*As previously stated, we had rewritten the manuscript to ensure that readers know the inclusion of the Northern vs Southern hemisphere is not only to analyse the results in the different hemispheres but also as a robustness test of our findings for the whole sample (page 4 line 107 and page 20 line 527). We also mention on page 6, line 145 and 28, line 710 of the manuscript that proximity and spatial distribution play roles in determining the effect of a disaster on happiness. Therefore, we maintain that the North vs South analysis will be of value to readers.*

5. **Finally I also remain concerned by the N observations (point 5 in my previous comments) notwithstanding the additional test the authors report.**

*As stated by Baltagi (2005), macro panels on countries or regions (thus N) with long time series (thus t) that do not account for cross-country dependence may lead to misleading inference. Additionally, it names different unit-root tests appropriate for testing cross-country independence.*

*Accounting for cross-country dependence turns out to be important and affects inference. Various examples of econometric studies argue that panel data will yield more powerful unit root tests than individual time series.*

*Therefore, we performed the most appropriate test for a long and unbalanced panel, the Im–Pesaran–Shin panel unit-root test (Im et al. 2003) and addressed it by introducing a lagged dependent variable (*${GNH}_{i-1,c}$*) in our model, as suggested by Greene (2010). Subsequently, we repeated the test and found no further evidence of autocorrelation. This means we have achieved stationarity, leading to robust estimations.*

*References*

*Baltagi, B. H. (2005). Econometric Analysis of Panel Data Third Edition, Chapter 12.*

*Greene, H. W. (2010). Econometric analysis. Chapter 11 in Models for Panel Data. Pearson Education Limited 2012.*

*Im, K., Pesaran, M., & Shin, Y. (2003). Testing for Unit Roots in Heterogeneous Panels. Journal of Econometrics, 115, 53–74.*
