# Supplementary material for: Reactions to macro-level shocks and re-examination of adaptation theory using Big Data
Source: PLoS One. 2024 Jan 31;19(1):e0295896. doi: 10.1371/journal.pone.0295896 (PMC10830054; doi:10.1371/journal.pone.0295896)
Supplement: Attachment — Submitted filename: Comments on PONE-D-22-19460r3.docx [file pone.0295896.s010.docx]

**Comments on PONE-D-22-19460r3 “Re-examining adaptation theory using Big Data: Reactions to External Shocks”**

Once again the authors have engaged in detail with my comments, for which I commend them.

We remain at odds regarding some key points where, as I said before, we will have to agree to disagree. I think it is for the editor to decide how to handle those differences.

Let’s go through those points again.

First, regarding the issue of treating lockdown as a ‘shock’. The authors point to papers in which others have referred to lockdown as a ‘shock’. I think the authors should spend more time incorporating these papers as a justification treating the lockdown as a shock and in using COVID as a right-hand side independent variable when, one might argue, this is the shock.

Second, I continue to question treating 2021 as a counterfactual period due to the dynamic and on-going effects of COVID. It’s very tricky interpreting results relative to a counterfactual period which is itself unstable. I think the authors should spend more time in the paper justifying this.

Third, the authors’ graphical depiction of the common trends is problematic. It is clear that there are dips in wellbeing prior to both lockdown and invasion. The problem is akin to the Ashenfelter Dip problem in that trends depart shortly before ‘treatment’ but in the Ashenfelter case there is a clear theoretical reason for this (unemployment as a qualifying condition for treatment). What is the explanation here. If this can be discussed that’d be helpful. I think the authors’ charts in the response letter are useful in identifying a pre-period where there were common trends so this should be used in estimating the dif-in-dif estimates. Was that done? Please make it clear.

I am happy to set aside the issues regarding the North/South issue and the N’s issue if the authors can address the above.
