# Supplementary material for: Reactions to macro-level shocks and re-examination of adaptation theory using Big Data
Source: PLoS One. 2024 Jan 31;19(1):e0295896. doi: 10.1371/journal.pone.0295896 (PMC10830054; doi:10.1371/journal.pone.0295896)
Supplement: Attachment — Submitted filename: Response to reviewer.docx [file pone.0295896.s011.docx]

FOURTH RESPONSE TO REVIEWER #2

**Manuscript ID PONE-D-22-19460R3**

**Re-examining adaptation theory using Big Data: Reactions to external shocks.**

**Once again the authors have engaged in detail with my comments, for which I commend them.**

**We remain at odds regarding some key points where, as I said before, we will have to agree to disagree. I think it is for the editor to decide how to handle those differences.**

*Thank you for your commendation. We have engaged in good faith with all your comments throughout the first three reviews. This is the fourth time we are responding to similar comments, and as you suggest, the editor should decide how to handle any remaining differences.*

**Reviewer #2's comments.**

**Let's go through those points again.**

1. **First, regarding the issue of treating lockdown as a 'shock'. The authors point to papers in which others have referred to lockdown as a 'shock'. I think the authors should spend more time incorporating these papers as a justification treating the lockdown as a shock and in using COVID as a right-hand side independent variable when, one might argue, this is the shock.**

*Thank you for your comment. Subsequently, we added a discussion showing the negative impact of lockdown (a shock) on, for example, health, the economy, mental health and loneliness under Section 2.1 on page 9. For ease, we copy the section below:*

*"* *Lastly, when it comes to the macro-level lockdown shock, Ohrnberger [25], Blanchflower and Bryson [26], Adams-Prassl et al. [27], Hamermesh [28], Brodeur et al. [29], Mahmud and Riley [30] and Sibley et al. [31] all successfully showed the negative impact of lockdown shock on, for example, health, the economy, mental health and loneliness. Ohrnberger [25] used difference-in-differences estimations on a longitudinal sample of 6,437 South Africans to investigate the causal effect of the lockdown shock on health and found that it significantly reduced health by 0.2 standard deviations. Blanchflower and Bryson [26] discussed how the US's economic shock occurred in response to policy efforts to contain the COVID-19 virus by going into lockdown in March 2020. Adams-Prassl et al. [27] used real-time US survey data to compare mental health before and after the introduction by state of lockdowns. They found that lockdown decreased mental health by 0.083 standard deviations and that changes in women's mental health mainly drove it. Hamermesh [28] examined data from the American Time Use Survey for 2012–2013 and used those data to simulate what would happen in lockdowns, given that this would result in people staying at home. He predicted that single people were less satisfied with life in running simulations than married people.*

*Brodeur et al. [29] used Google Trends data to test whether the lockdowns implemented in Europe and the US led to changes in well-being-related topic search terms. Their results suggest that the lockdown may have severely affected people's mental health. Mahmud and Riley [30] provided evidence of the impact of the COVID-19 lockdown shock on the economic and well-being of a sample of households in rural Uganda. Their results were among the first to show a significant negative impact of the lockdown on a rural population. Sibley et al. [31] found that lockdown regulations slightly increased people's sense of community and trust in institutions. However, on the other hand, they cautioned that there would be longer-term challenges to mental health since anxiety and depression levels were up post-lockdown."*

*Regarding your comment on using a COVID-19-related covariate as a right-hand side independent variable, we have added the following under section 3.3 at the bottom of page 16 and at the top of page 17, where we discuss the selection of covariates.*

*"* *The number of new COVID-19 cases. We use the lagged number of new COVID-19 cases per million to control for the evolution of the pandemic (Hale et al. [58]). Since lockdown is the shock we are investigating, it is imperative that we incorporate a COVID-19-related variable as a control, given the intricacies associated with isolating the unique impact of lockdown on well-being. A failure to control for the effects of COVID-19 could undermine the validity of attributing observed effects solely to lockdown measures rather than the broader influence of the COVID-19 pandemic itself. Here, we follow the work of Rossouw et al. [35], Brodeur et al. [29], Greyling et al. [33] [34] and Sarracino et al. [37], who included COVID-19-related variables as controls."*

2. **Second, I continue to question treating 2021 as a counterfactual period due to the dynamic and on-going effects of COVID. It's very tricky interpreting results relative to a counterfactual period which is itself unstable. I think the authors should spend more time in the paper justifying this.**

*We have discussed the treatment of 2021 as a counterfactual at length (in three different paragraphs) and included references to statistical papers on counterfactuals, supporting and validating our choice of 2021 as a counterfactual. For your reference, the first discussion is under section 3.1 on page 11, but we copied it below for ease.*

*"* *In the analyses, we use two macro-level shocks, namely the first lockdown during COVID-19 and Ukraine's invasion, and compare the pre- and post-event days. The lockdown shock was in 2020, and the invasion in 2022. In choosing our counterfactual period, we follow the pioneering work of* *Bennet [52], Lewis [53] [54] [55], and Menzies and Beebee [56]. In their work, they state that a counterfactual should be closer to actuality than another if the first resembles the actual world more than the second does. Therefore, as a counterfactual period, we use the same days in the year 2021. Another option was using 2019, but 2019 was not 'closer to the actuality' of 2020 and 2022. Simply put, 2021 is a better counterfactual than 2019 because 2021 shares more commonalities with the years of the shocks than 2019. For example, during 2019, COVID-19 or COVID-19-related shocks, such as lockdowns, which had further socio-economic consequences, did not exist. Using 2019 as a counterfactual is not an option since it could not resemble the actual world of 2020 and 2022 because COVID-19 changed the world, and we can never revert back to the times before COVID-19. We assume that 2021 reflects the 'new normal' in which people have accepted COVID-19 as a reality and part of their daily lives."*

*We also refer to our counterfactual being closer to the actuality of 2020 and 2022 in section 3.4.1 on page 17 and again at length on page 19. We copy it below for ease.*

*"* *We also use context-specific knowledge to argue that 2021 should be selected as the counterfactual since it is similar to the years of the events. COVID-19 occurred in the selected periods; therefore, we are comparing very similar periods since there was no COVID-19 prior to 2020. This means the parallel trends assumption is likely more credible for 2020/2021 and 2021/2022 than if you were comparing periods quite different from one another, i.e., 2019/2020. Considering 2020/2021 and 2021/2022, we also find many similarities between the time periods, thus supporting the parallel assumption. These similarities include i) the seasons are similar, and ii) many public holidays in the countries are on the same date in 2020 and 2021 and in 2022 and 2021 – often capturing higher happiness levels on these days. We also considered other factors, including higher unemployment rates (we saw some recovery late in 2022, but it does not affect our analyses since we analysed the data in February 2022, and as such, the recovery was still not apparent). Other similarities are that GDP growth decreased, inflation rates increased, and consumer spending decreased; these trends were visible in the year of the shocks and the counterfactual year. Furthermore, as mentioned in section 3.1, the choice of the 2021 period makes logical sense. In all time periods, COVID-19 is a reality, not in 2019."*

*Therefore, you can see that our discussion is sufficiently extensive in the manuscript. We maintain that any addition to these discussions will be non-informative and repetitive, something we wish to avoid.*

3. **Third, the authors' graphical depiction of the common trends is problematic. It is clear that there are dips in wellbeing prior to both lockdown and invasion. The problem is akin to the Ashenfelter Dip problem in that trends depart shortly before 'treatment' but in the Ashenfelter case there is a clear theoretical reason for this (unemployment as a qualifying condition for treatment). What is the explanation here. If this can be discussed that'd be helpful.**

*Thank you for these observations and the reference to the Ashenfelter dip – this is indeed very similar to what we observe during shocks. With the Ashenfelter, it is mentioned that dips might be observed pending the treatment, perhaps due to some anticipation. Also, it indicates that there might be some adaptation before treatment, so the effect can often be underestimated. In our study, we explained the reasons for the dips and previously included the explanations with the event study results (Section 4.3 on page 27). However, we now also mention it in the DiD result section (Section 4.2 on page 23) and cross-reference it to the event study results. We also refer to the fact that what we see is similar to the Ashenfelter dip to explain our observations further.*

4. **I think the authors' charts in the response letter are useful in identifying a pre-period where there were common trends so this should be used in estimating the dif-in-dif estimates. Was that done? Please make it clear.**

*In our previous response document, we had the below paragraph when we discussed the common trends assumption:*

*"Furthermore, we plot graphs with an extended time period before the shocks. You will notice that though the trends are not perfectly parallel, the trends before the lockdown and invasion show they are closely related. In addition, after some time lapse after the shocks, we seem to find parallel trends, thus strengthening our assumption. Please see below."*

*We apologise; this is our mistake, and our choice of words was incorrect. We should have written that we expanded the scale of the graphs so that the parallel trends were more visible, meaning the estimations were unchanged. We added the graphs below (also included in the previous response) to the paper with the expanded scales and removed the previous graphs.*

5. **I am happy to set aside the issues regarding the North/South issue and the N's issue if the authors can address the above.**

*Thank you. Please see all the responses to your previous comments, which we addressed in points #1 to #4.*
